# The Blocking Effect of Clay in Groundwater Systems: A Case Study in an Inland Plain Area

**DOI:** 10.3390/ijerph15091816

**Published:** 2018-08-22

**Authors:** Liting Xing, Linxian Huang, Yi Yang, Junxiang Xu, Wenjing Zhang, Guangyao Chi, Xinyu Hou

**Affiliations:** 1 School of Water Conservancy and Environment, University of Jinan, Jinan 250022, China; stu_xinglt@ujn.edu.cn (L.X.); shandongchiguangyao@hotmail.com (G.C.); hxy1434672208@outlook.com (X.H.); 2 Engineering Technology Institute for Groundwater Numerical Simulation and Contamination Control, Jinan 250022, China; 3 Jinan Water Conservancy Construction Survey Design Research Institute, Jinan 250014, China; yy18363034015@hotmail.com; 4 Shandong Provincial Bureau of Geology & Mineral Resources, Jinan 250013, China; xjx13969153372@hotmail.com; 5 Shandong Yu Qiang Geological Resource Exploration and Development Co., Ltd., Taian 271000, China; zwj18363035945@hotmail.com

**Keywords:** clay, adsorption isotherm equations, blocking effect, groundwater chemical composition, inland plain area

## Abstract

In order to increase understanding of the hydrogeochemical effects that influence changes in the quality of salt water, we investigated the distribution of saline and fresh water in an inland plain area and, in particular, the scarcity of fresh water resources. Taking the inland plain in Jiyang County as a specific case study, samples of undisturbed clay and underground saline water from different depths were collected to examine hydrogeological changes. A wide variety of methods was used to analyze the blocking effect of clay on the chemical characteristics of the groundwater. These include real-time monitoring for field water quality, tests for isothermal adsorption, a factor analysis model, physiochemical analysis, and correlation analysis. Our results show that the optimal adsorption isotherm of clay for Na^+^, Ca^2+^ and Mg^2+^ in groundwater conform to the established Henry and Langmuir equations for adsorption isotherms. The influence of clay mineral types and content on the blockage of Na^+^, Ca^2+^ and Mg^2+^ in groundwater samples were evident at different depths, with the clay adsorption capacity increasing in line with increases in the clay mineral content. Clay at different depths was found to have the strongest blocking effect on Na^+^ in groundwater, being systematically greater than its effect on Ca^2+^ and Mg^2+^. It is believed that the blocking effect of clay has an important influence on the hydrochemical zoning of groundwater in inland plains and the formation of saline water in groundwater systems. This study therefore provides concrete evidence in support of this supposed effect.

## 1. Introduction

Clay has a negative charge under normal or salt water conditions. In order to maintain its neutrality, clay adsorbs cations in groundwater. As Na^+^, Ca^2+^ and Mg^2+^ are the most common cations in groundwater, when the groundwater concentration reaches a certain value, they can be blocked by clay [1]. 

The strength of the blocking effect is not only related to the properties of the clay itself [2] and the hydrodynamic characteristics of the groundwater flow system [3,4], but also to the degree of mineralization of the groundwater [5,6]. In general, the ability of clay to block Na^+^, Ca^2+^ and Mg^2+^ in groundwater depends on the nature and mass fraction of the clay minerals. The larger the specific surface area of the clay minerals, the greater their adsorption and ability to block cations [7,8,9]. Clay particles with a large specific surface area form a double electrical layer on the surface of the clay when Na^+^, Ca^2+^ and Mg^2+^ interact with groundwater. The pH value of the groundwater, however, causes a change in the properties of this double electrical layer, which affects the adsorption capacity of the clay particles for Na^+^, Ca^2+^ and Mg^2+^ [8,9,10,11,12]. So, to give an example, once the mass concentration of Na^+^ (Ca^2+^ and Mg^2+^) in the groundwater exceeds 200 mg/L, the clay will begin to absorb the Na^+^ [13,14]. A large number of studies have also demonstrated that the retarding strength of clay on underground salt water decreases when there is an increase in effective porosity or the hydraulic gradient. Once the hydraulic gradient passes a certain threshold, shallow salty water will flow into deep freshwater [15,16,17,18]. During this leakage process, the retardation coefficient related to the strength of the clay blocking effect becomes inversely proportional to its own permeability coefficient [19,20,21]. 

There is a large body of literature dedicated to the water chemistry mechanisms associated with saline water leakage. Various methods have been used to examine the system in more detail, including factor analysis [22], the ion ratio method [23], isotope tests [24], trace element tests [25], and so on. Factor analysis is the principal approach used for the comprehensive evaluation of multi-factor systems. Factor analysis is able to fully encompass overlaps in information between different evaluation indicators. It is also able to bring about a comprehensive reduction of high-dimensional variables by retaining original information to the maximum extent. The weight of each index can then be objectively determined, thus offsetting any subjective arbitrariness. 

There are many ways to study the blocking effect of clay on groundwater chemical components, principal amongst them being high-pressure permeation tests [26] and adsorption isothermal tests [27]. Adsorption isotherm experiments are relatively straightforward, with a shorter duration than pressure permeation tests and the adsorption isotherm equation and test data have a higher degree of fit. Some scholars have found a particular value in using a Langmuir formula to describe the adsorption process. This is because it defines an adsorption limit at a given adsorption site that will meet certain specified criteria, then estimates the total adsorption capacity of the clay for different adsorbates. This provides a solid foundation for the study of the clay adsorption mechanism. The process for clay adsorption of groundwater chemical components is mainly linear adsorption (what is known as the Henry model). As an example, the adsorption isotherms of clay for niobium and ammonia nitrogen conforming to the Henry model will have retardation coefficients of 2523.36 and 60.87, respectively [28,29]. For monolayer chemical adsorption, the adsorption isotherms of clay for organophosphorus and Mn^2+^ will conform to the Langmuir model [30,31,32]. For physical adsorption, the clay adsorption of ammonium ions [33,34] will conform to what is known as the Freundlich model [18,21]. On the basis of these various models, a significant number of previous investigations have shown that clay has a blocking effect on pollutants and have sought to highlight some of the key factors involved. However, there is a notable lack of discussion regarding the mechanisms whereby clay can serve to block Na^+^, Ca^2+^ and Mg^2+^ in groundwater.

The presence of finer sediment particles in inland plains, the high proportion of clay minerals, and stagnant groundwater runoff all contribute to a vertical alternation of saline and fresh water, with hydrochemically characteristic horizontal and vertical zoning [35]. Over the past few decades, excessive exploitation of deep fresh water in inland plains has caused shallow saline water to flow into deep aquifers, which in turn has led to their salinization. In that case, controlling and preventing salt water from continuing to invade deep fresh water in inland plains has become important to the sustainable use of groundwater resources. As an important aspect of the saline water leakage system in inland plains, clay plays a key role in the distribution of brackish water and the downward invasion of underground salt water. In order to analyze the influence of adsorption and the blocking effect of clay on the chemical components of groundwater, we used geological drilling and pumping tests to collect undisturbed clay and water samples from different depths in the inland plains of Jiyang county in China. Real-time monitoring of field water quality, adsorption isotherm experiments, factor analysis and correlation analysis were all then used to investigate the mechanisms whereby clay can serve to block major common cations. This, in turn, will serve to increase our understanding of the hydrogeochemical effects and development processes that influence the distribution of saline water. 

## 2. Materials and Methods 

### 2.1. Overview of the Study Area

**Figure 1:**
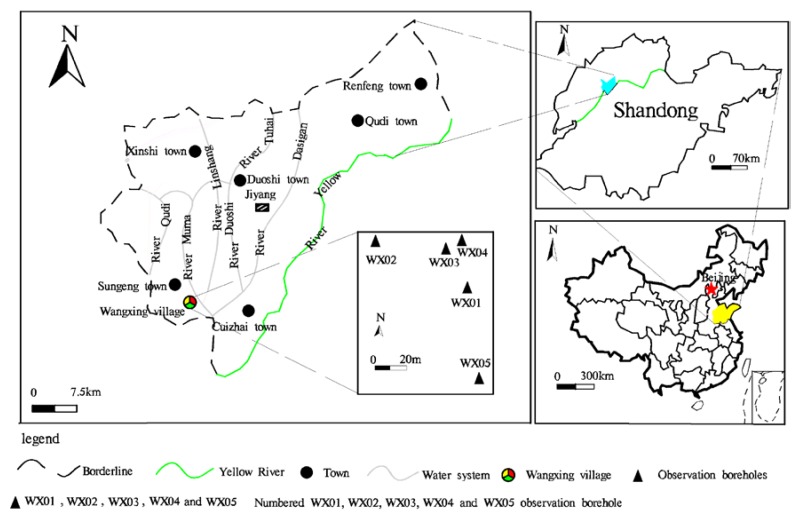
Location of the study area.

**Figure 2:**
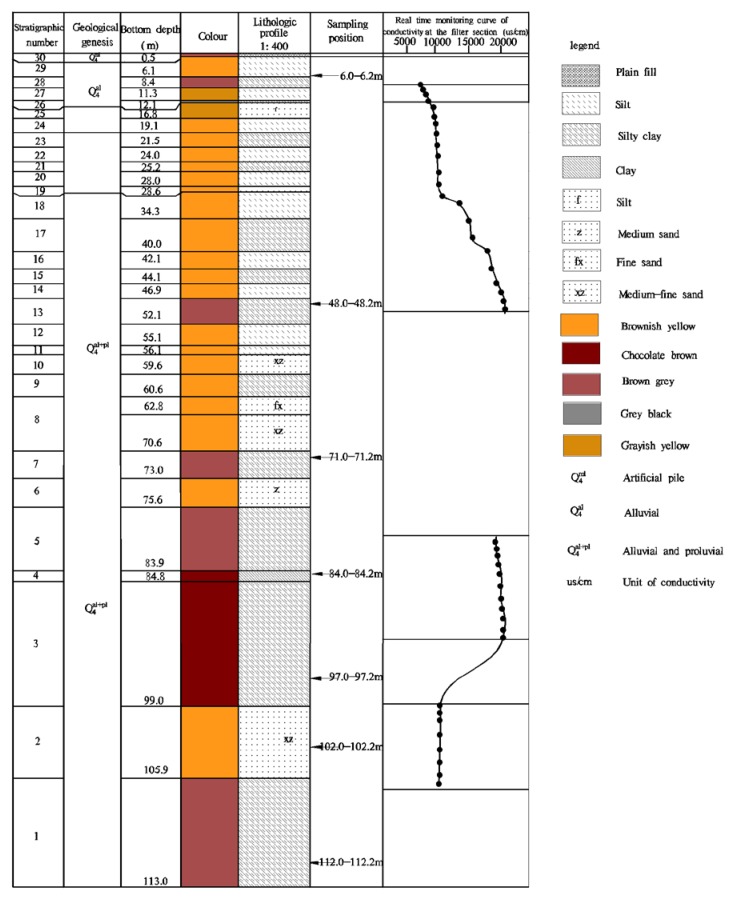
Stratigraphic histogram for depths of 0–113 m.

The research took place in Jiyang County, Shandong Province, an inland region of China (Figure 1) with a warm temperate continental monsoon climate. The average annual temperature is 14.3 °C, the average annual rainfall is 665.7 mm and the average annual evaporation is 1700.2 mm. So its four seasons are distinct. The study area is mainly covered with loose Quaternary deposits, the other deposits being Neogene, Permian, Carboniferous, and Ordovician (Figure 2). The 500 m of shallow water in the area can mostly be found in the pores of the Quaternary and Neogene loose deposits. Salt water has a wide distribution in the mainland. Here, the degree of salinity for the salt water in the vertical direction, first of all increases, then decreases with an increase in depth. The change range is 5–9 g/L. In the plane, the water chemistry is varied and its composition is complex. The Total Dissolved Solids (TDS) is generally 2~5 g/L. The salt water is located in the runoff stagnation area between the ancient rivers.

### 2.2. Sample Collection

After taking into account current field hydrogeological conditions and site constraints, Wangxing Village, Sunjing Town was selected as a typical location for investigating salt water in the inland plains. Four boreholes with a range of 65 m × 45 m were selected to serve as the water level and water quality measurement boreholes (see Figure 1). The boreholes were numbered WX01, WX02, WX03 and WX04, with No. 4 being an existing borehole. Water filtration pipes for hydrogeological observations were placed within the boreholes at depths of 8–12 m, 79–94 m, 98–107.9 m, and 27–49.2 m. The lithology for the hydrogeological observation borehole WX01 was silty soil and silty clay soil. For borehole WX02, the lithology was silty clay and cohesive soil. The lithography for borehole WX03 was silty clay and clay, whilst the lithology for borehole WX04 was dominated by silt, fine sand, and medium sand. The basic parameters for the hydrogeological drillings are shown in Table 1.

The physical properties of the collected undisturbed soil (grain specific gravity, water content, density, and void ratio) were tested using a geotechnical test. The particle size of the soil was determined using a particle size sedimentation analysis test, and the X-Ray Powder Diffusion (XRD) analysis software, MDI Jade (San Francisco, CA, USA), was used for phase analysis and quantitative analysis of the clay. Adsorption and the blocking effect of the clay on Na^+^, Ca^2+^, and Mg^2+^ in the groundwater at different depths were determined by means of isothermal adsorption experiments. In accordance with technical regulations, undisturbed soil samples from depths of 6.0–6.2 m, 48.0–48.2 m, 51.0–51.2 m, 71.0–71.2 m, 84.0–84.2 m, 97.0–97.2 m, 102.0–102.2 m and 112.0–112.2 m were collected. The void ratio and clay mineral content of the undisturbed soil samples were measured, with the void ratio being between 0.496 and 0.849 and the clay mineral content being within the range of 21–46%. The coefficient of variation was 24.49% (see Table 2). The soil samples (3000 g) used for sorption isotherm tests were collected from each depth sample, from which the debris was removed. The samples were air dried in a cool ventilated area, sieved, ground, fully mixed, bottled and retained for adsorption tests.

Water samples for analysis were taken from the four hydrological observation boreholes, sealed and stored [36]. Sample preparation was completed within 7 days of returning the samples to the laboratory. The milliequivalent percentage of water chemical composition is shown in Table 3. The hydrochemistry type for the groundwater in boreholes WX01, WX03 and WX04 was ClSO_4_–NaMg. The hydrochemistry type for the groundwater in borehole WX02 was ClSO_4_–MgNa.

### 2.3. Real-Time Field Groundwater Quality Monitoring 

Groundwater from the water filtration sections of the four hydrological observation boreholes was analyzed for water temperature (T), pH and electrical conductivity (EC) using an Aqualtroll600 multi-parameter water quality detector (In-Situ, Los Angeles, CA, USA). The test accuracy was ±0.1 °C, ±0.1 and 1 ± 0.1 μS/cm for the three physiochemical indicators, respectively. As conductivity reflects the number of ions in the water, this indicator can be used to characterize changes in the degree of mineralization [37]. 

### 2.4. R-Type Factor Analysis

Numerous studies have suggested that R-factor analysis is the best approach to establishing water chemistry [17], so this was used to infer the hydrochemistry for the groundwater at different depths. The test indicators were Na^+^, Ca^2+^, Mg^2+^, HCO_3_^−^, Cl^−^, SO_4_^2−^, total hardness, pH and EC. After correlation analysis and factor analysis using SPSS 10.0 (San Francisco, CA, USA), the correlation coefficient matrix for each index was obtained. A main component analysis method was used to extract the eigenvalues and the main factor was selected. In order to accurately interpret the meaning represented by the main factor, a maximum variance rotation method was used to rotate the index matrix.

### 2.5. Indoor Adsorption Isothermal Test and Determination of the Retardation Factor

#### 2.5.1. Indoor Adsorption

Indoor adsorption isothermal tests were performed using a batch method [38]. Clay samples from depths of 6, 48, 51, 71, 84, 97, 102 and 112 m were selected, washed and dried with ultra-pure water. The test water samples were diluted to 10 solutions with a gradient of conductivity of 2000, 4000, 6000, 8000, 10,000, 12,000, 14,000, 16,000, 17,000, and 188,400 us/cm. First of all, ten samples of 30 g of clay were transferred to a 250 mL stoppered erlenmeyer flask. The above-mentioned solutions were then added separately. The temperature of the solution was maintained at 18 ± 1 °C throughout the test and the pH was 7.5 ± 0.5. The erlenmeyer flask was then placed in a constant-temperature shaker bath (SHZ-82A, Shimadzu company, Tokyo, Japan) to oscillate for 3 h. Once adsorption had reached a point of saturation, samples were extracted. These samples were centrifuged at 4000 r/min, then filtered through a 0.45 μm microporous membrane. The filtrate was analyzed using a PXSJ-216F ion meter (Shanghai Yidian Scientific Instrument Company, Shanghai, China) to determine the mass concentration of Na^+^ in the samples. Then a Shimadzu-AA7000 (Shimadzu company, Tokyo, Japan.) atomic absorption spectrophotometer was used to measure the concentration of Ca^2+^ and Mg^2+^ in the filtrate. The sample analysis was repeated three times for each solution. Three hundred and seven groups of valid data were obtained by the indoor adsorption isothermal test. Adsorption isotherm analysis was then performed on the valid data.

#### 2.5.2. Determination of the Retardation Factor

The adsorption capacity of the adsorbent (clay) for the adsorbates (Na^+^, Ca^2+^ and Mg^2+^; *q_e_*) was calculated as follows [39]:

(1) $$q_{e}=\frac{\left(C_{0}-C_{e}\right)\;V}{M}$$

The adsorption isotherm indicates the relationship between the adsorbent surface and the adsorbate in the solution. The isotherm was fitted using the Langmuir isotherm Equations (2) and the Henry isotherm Equations (3). The effectiveness of the fittings were evaluated using the determination coefficient *R*^2^ [39].

(2) $$q_{e}=q_{m}\frac{K_{L}C_{e}}{1+K_{L}C_{e}}$$

(3) $$q_{e}=K_{d}C_{e}$$

For this analysis, the linear distribution coefficient and the effective porosity of the clay were calculated first (4), then the retardation coefficient (*R_d_*) (5) [40]:

(4) $$n=\frac{e_{0}}{1+e_{0}}$$

(5) $$R_{d}=1+\frac{1-n}{n}K_{d}$$

In the above Equations (1)–(5), *C*_0_ is the initial mass concentration of the adsorbate in the solution (mg L^−1^); *C_e_* is the adsorption equilibrium mass concentration (mg L^−1^) of the adsorbate in the solution; *q_e_* is the equilibrium adsorption capacity of the adsorbent (mg kg^−1^); *V* is the volume of the solution (L); *M* is the mass of the adsorbent (kg); *q_m_* is the maximum adsorption capacity of the adsorbent in the Langmuir adsorption equation (mg kg^−1^); *K_L_* is the adsorption equilibrium constant (L mg^−1^) of the adsorbent in the Langmuir adsorption equation; *K_d_* is the partition coefficient in the Henry equation (L kg^−1^); *e*_0_ is the void ratio; *n* is the effective porosity; and *R_d_* is the retardation coefficient.

## 3. Results and Analysis

### 3.1. The Effects of Clay Adsorption on the Chemical Characteristics of Groundwater

Three main factors (F1–F3) were identified through the factor analysis model (Table 4 and Table 5). The first main factor (F1) influencing groundwater in the aquifer at a depth of 8.0–12.0 m included Mg^2+^, Cl^−^, SO_4_^2−^ and total hardness, with a variance contribution rate of 64.164%. The main factor load values of the four hydrochemical indicators were all above 0.8. The second main factor (F2) was mainly determined by the pH, with a variance rate of 31.235%. This result indicates that F1 and F2 reflect the effects of leaching and cation exchange adsorption, together with the influence of an acid-based evolution of the chemical composition of the groundwater during runoff.

For the groundwater at a depth of 13.0–49.2 m, F1 included seven water chemistry indicators (Na^+^, Ca^2+^, Mg^2+^, Cl^−^, SO_4_^2−^, total hardness and EC), with a variance contribution rate of 79.446%. The main factor load value was above 0.9 and EC and F1 had a negative correlation. In this case, F2 was mainly determined by HCO_3_^−^ and the pH and its variance contribution rate was 16.941%. Both the load values of the two water chemistry indicators in F2 were above 0.5 and the pH was negatively correlated with F2. This indicates that F1 and F2 reflect the effects of mixing, cation exchange adsorption, carbonic acid evolution and the acid-based evolution of the chemical composition of the groundwater. 

F1 in the groundwater aquifer at a depth of 79.0–94.0 m included Na^+^, SO_4_^2−^, pH and EC. The variance contribution rate was 34.943% and the values of the four water chemical indicators in F1 were above 0.6. F2 was mainly determined by Mg^2+^ and Cl^−^, with a variance contribution rate of 22.397%. The load values of the two water chemical indicators were both greater than 0.7 and there was a negative correlation between Cl^−^ and F2. The third main factor (F3) in this aquifer was mainly determined by Ca^2+^, with a variance contribution rate of 13.456%. The water chemistry indices in the main factor load values were all greater than 0.6. In this case, F1, F2 and F3 reflect the effects on the chemical composition of the groundwater of concentration, acid-based evolution, leaching, ion exchange adsorption and dissolution/precipitation of calcite and gypsum. 

Groundwater from an aquifer depth of 98.0−107.9 m recorded an F1 that was dominated by Ca^2+^, SO_4_^2−^ and total hardness, with a variance contribution rate of 54.647%. The three water chemical indicators in the main factor load value were all greater than 0.7. F2 was mainly determined by Ca^2+^, HCO_3_^−^ and EC, with a variance contribution rate of 14.607%. The water chemical index was greater than 0.45 and there was a negative correlation between Cl^−^ and F2. F3 was mainly determined by EC, its variance contribution rate being 12.138%. The water chemistry index in the main factor load value was greater than 0.7. Here then, F1, F2 and F3 are reflecting the effects on the chemical composition of the groundwater of dissolution/precipitation, leaching, ion exchange adsorption and the mixing of calcite and gypsum.

### 3.2. The Adsorption Model of Na^+^, Ca^2+^ and Mg^2+^ in Groundwater by Clay

**Figure 3:**
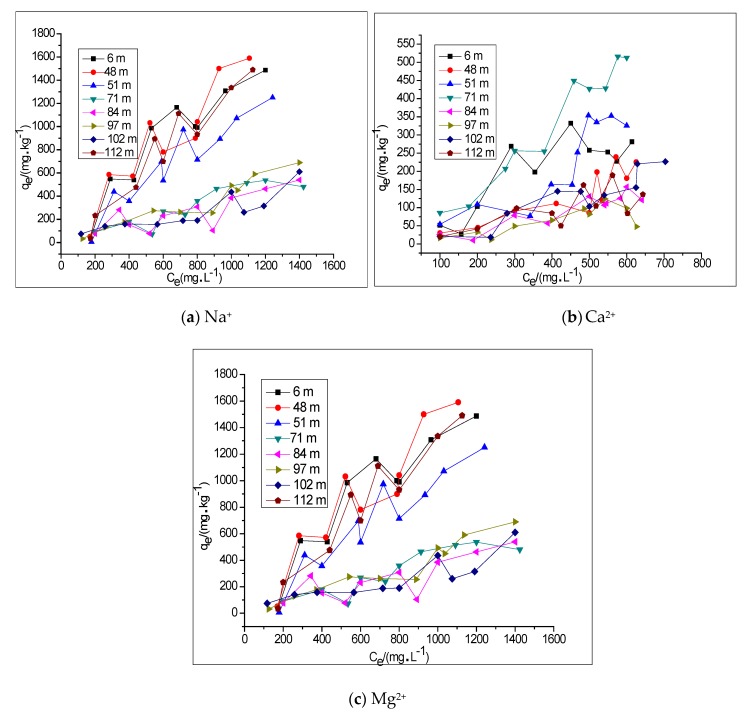
Adsorption isotherm of clay for (**a**) Na^+^, (**b**) Ca^2+^ and (**c**) Mg^2+^ in groundwater at different depths. Note: *C_e_* is the adsorption equilibrium mass concentration (mg L^−1^) of the adsorbate in the solution; *q_e_* is the equilibrium adsorption capacity of the adsorbent (mg kg^−1^).

The adsorption characteristics and adsorption isotherms of clay for Na^+^, Ca^2+^ and Mg^2+^ in groundwater (Figure 3) show that the adsorption capacity increases in line with an increase in the concentration of Na^+^, Ca^2+^ and Mg^2+^. It can also be seen that, as the concentration of the ions increases, the adsorption capacity continues to increase. This indicates a fast-paced process of change. The maximum adsorption capacity of clay for Na^+^, Ca^2+^ and Mg^2+^ in groundwater corresponds to the maximum adsorption capacity obtained from the Langmuir equation. The deviation was only 5–15% and the adsorption isotherm could be fitted to the Henry equation. As the adsorption isotherms of Na^+^, Ca^2+^ and Mg^2+^ for clay have a relatively high correlation (see Table 6) and conform to the Henry and Langmuir equations, this indicates either monomolecular chemical adsorption or linear adsorption processes.

### 3.3. Clay Adsorption Affecting the Formation of Na^+^, Ca^2+^ and Mg^2+^ in Groundwater 

**Figure 4:**
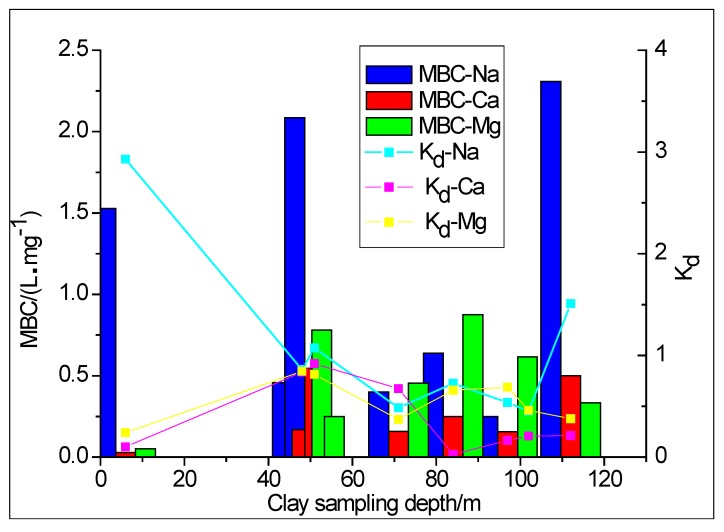
The maximum buffering capacity (MBC) trend and the linear distribution coefficient of clay for Na^+^, Ca^2+^ and Mg^2+^ in groundwater. Note: *K_d_* is the partition coefficient in the Henry equation (L kg^−1^).

**Figure 5:**
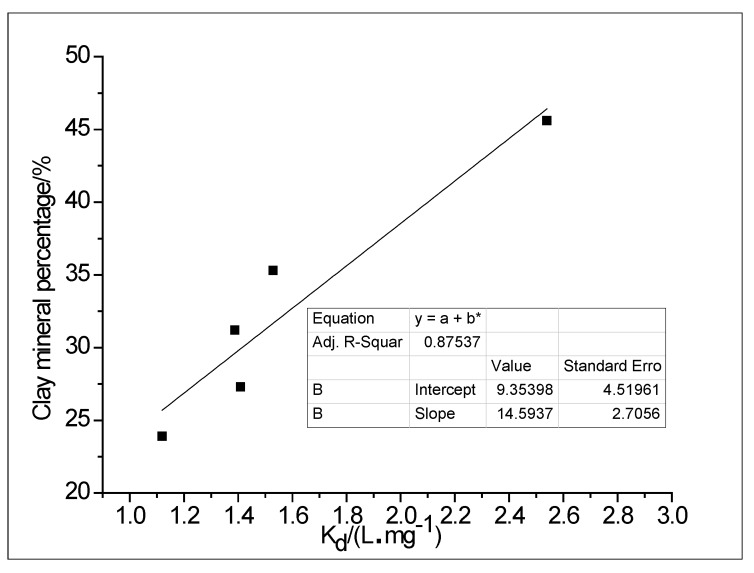
Correlation between the linear distribution coefficient at different depths of clay and the total content of clay minerals.

The maximum buffering capacity (MBC) of clay is the product of the maximum adsorption quantity (*q_m_*) and the adsorption equilibrium constant (*K_L_*) in the Langmuir sorption isotherm equation. The linear distribution coefficient (*K_d_*) in the clay adsorption process is the ratio of the conventional ion concentration in the clay to the conventional ion concentration in the groundwater. These factors all reflect the adsorption capacity of clay. Based on the calculation and analysis of MBC and *K_d_* (see Figure 4), the order of adsorption of clay on conventional ions in groundwater at a depth of 6 m and 48 m was: Mg^2+^ > Na^+^ > Ca^2+^. At depths of 51 m and 112 m the order was: Na^+^ > Ca^2+^ > Mg^2+^; and at depths of 71 m, 84 m, 97 m and 102 m the order was: Na^+^ > Mg^2+^ > Ca^2+^. The correlation between the linear distribution coefficient for the different depths of clay and the total content of clay minerals was as high as 0.8754 and the linear distribution coefficient increased with an increase in the clay mineral content (Figure 5). The difference in adsorption capacity may therefore be due to differences in the amount of illite and montmorillonite, the main clay minerals that were found to differ at different depths, together with differences in the type of ion exchange and cation exchange adsorption capacity [41]. To sum up, the adsorption ability of clay for Na^+^ in groundwater is relatively strong with a certain controlling effect on the formation of chemical components in the groundwater, whilst the clay adsorption capacity is affected by the quantity of clay minerals.

### 3.4. The Blocking Effect of Clay on the Vertical Migration of Na^+^, Ca^2+^ and Mg^2+^ in Groundwater 

According to the retardation coefficient of clay for Na^+^, Ca^2+^ and Mg^2+^ in groundwater (see Table 7), the clay at certain depths has a different retarding strength for the different conventional ions in the groundwater. The order of blocking strength of clay on conventional ions at depths of 6 m, 48 m and 112 m was: Na^+^ > Mg^2+^ > Ca^2+^. At depths of 51 m and 71 m, the order was: Na^+^ > Ca^2+^ > Mg^2+^; and at depths of 84 m, 97 m and 102 m the order of blocking was: Mg^2+^ > Na^+^ > Ca^2+^. Different depths of clay have different degrees of blocking effect on Na^+^ (Ca^2+^ and Mg^2+^) in the groundwater. The retarding strength of clay for Na^+^, Ca^2+^ and Mg^2+^ in the groundwater decreased from 6 m to 102 m to 112 m. From depths of 6 m to 97 m to 112 m, and from 6 m to 102 m to 112 m, a trend of increase-decrease was recorded. 

**Figure 6:**
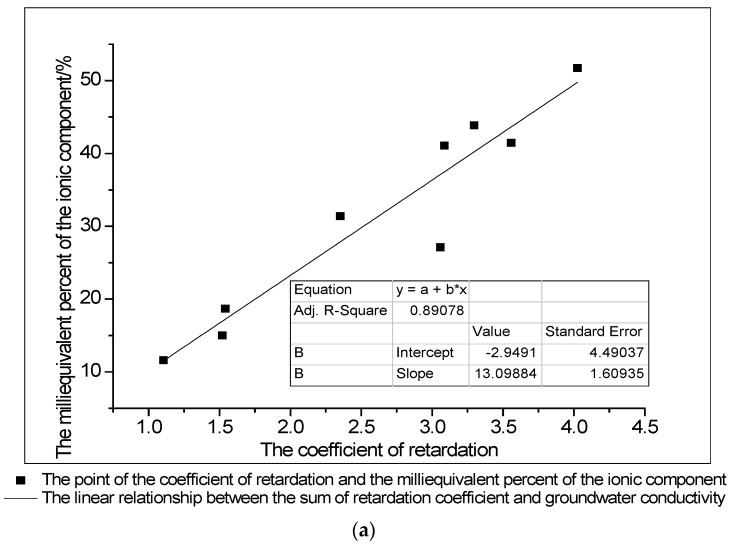

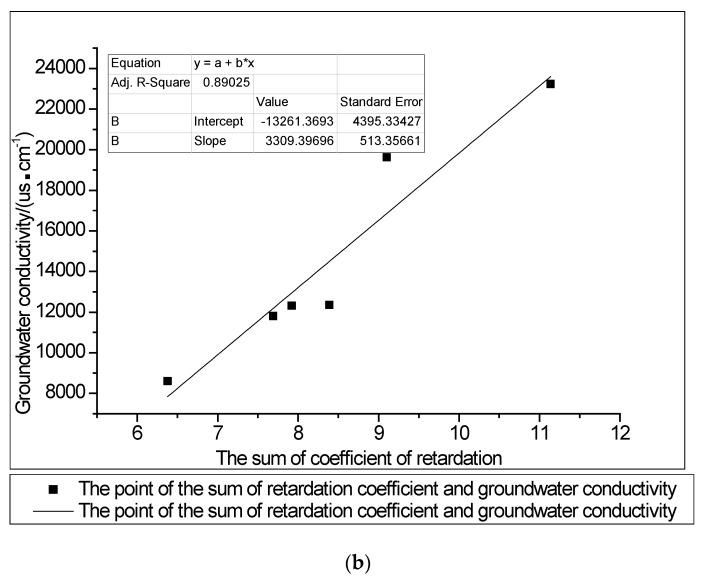
(**a**) Correlation between the coefficient of retardation and the milliequivalent percentage of the ionic components; (**b**) Correlation between the sum of retardation coefficients and groundwater conductivity.

According to the real-time monitoring of groundwater quality from 31 December 2016 to 4 March 2017, the groundwater water quality in each hydrological observation borehole was relatively stable. The average conductivity in the four boreholes, at groundwater depths of 8.0–12.0 m, 13.0–49.2 m, 79.0–90.0 m and 98.0–107.9 m, was 8801.82 μS/cm, 14,603.57 μS/cm, 18,734.42 μS/cm and 11,314.42 μS/cm, respectively. The correlation coefficient of the retardation coefficient of clay for Na^+^, Ca^2+^ and Mg^2+^ in groundwater and the milliequivalent percentage of Na^+^, Ca^2+^ and Mg^2+^ in equivalent depths of groundwater were as high as 0.89078 (Figure 6a). The content of Na^+^, Ca^2+^ and Mg^2+^ in groundwater increases as the clay blocking strength increases. The correlation between the sum of the retardation coefficients of clay for Na^+^, Ca^2+^ and Mg^2+^ in groundwater and the corresponding depth’s electrical conductivity observed in real-time was as high as 0.89025 (Figure 6b). This finding indicates that the larger the sum of the retardation coefficients, the higher the conductivity. Thus, we can observe that the blocking effect of clay has an important influence on the vertical migration of Na^+^, Ca^2+^ and Mg^2+^ in groundwater, the type of water chemistry and water quality zoning. This further confirms that clay has a controlling effect on the downward movement of salt water.

## 4. Discussion

(1) It is a consensus that illite and montmorillonite in clay can adsorb certain ionic components in groundwater, such as Cu^2+^ [42], Pb^2+^, Zn^2+^, Cd^2+^, Cr^3+^ [43], cadmium [44], Na^+^, Ca^2+^ and Mg^2+^ [45,46]. These minerals, affecting the chemical properties of groundwater, are contained in the clay of this area, which is consistent with the conclusions of the factor analysis methods in this paper.

(2) Former researchers concluded different adsorption models, to name a few, Henry model, namely linear adsorption; Langmuir model, i.e., mono-layer chemical adsorption; Freundlich model, namely physical adsorption. The adsorption isotherm curve of clay for anthracene and ammonia nitrogen conforms to the Henry model [28,29]. The process of clay adsorption of organic phosphorus and Mn^2+^ in groundwater is consistent with the Langmuir model [30,31,32]. The adsorption isotherm curve of clay for ammonium ions accords with the Freundlich model [33,34]. It can be seen that different ions have different models. Therefore, it is reasonable to say that the adsorption model of clay in this area is different for Na^+^, Ca^2+^ and Mg^2+^ in groundwater.

(3) Different depths of clay have different degrees of retardation on Na^+^ (Ca^2+^ and Mg^2+^) in groundwater, and with the increase of depth, the block strength of clay in groundwater decreases, which is related to the nature of the clay and its age of sedimentation. The newly deposited clay has strong retardation ability. The aged clay deposited in geological history can be traced back to a long time, and the retardation ability is reduced due to the secondary fracture caused by clay consolidation. Within the tens of thousands of square kilometers of the North China Plain, such as Jiyang, Dezhou, Cangzhou, Tianjin, and Dongying, different groundwater types exist at different depths [47,48,49]. In the vertical direction, there is shallow fresh water—middle salt water—deep fresh water within the depth of 600 m [33]. The clay of less than 200 m is relatively new so that the block strength is stronger, and the groundwater upward conductivity is significantly zonal. The clay deposits at a depth of 300–600 m are ages ago, but the difference between 300–600 m is not salient, and only deep fresh water is present. Therefore, the retardation of clay is one of the factors affecting the vertical zoning of groundwater hydrochemistry.

## 5. Conclusions

In recent years, the downward intrusion of shallow saline groundwater in inland plains (especially the North China Plain) has disabled a large number of deep aquifer extraction wells because of the salinization of deep groundwater. Control and prevention of this downward intrusion is therefore key to the sustainable use of groundwater resources in the inland plains. Clay can play a critical role in controlling the downward intrusion of shallow saline groundwater, so understanding the mechanism involved in this downward intrusion is a fundamental prerequisite for being able to exercise such control. There is large body of literature dedicated to the blocking effects of clay, but most of it focuses on the factors affecting the clay blocking coefficient, such as effective porosity, permeability, concentration of seepage fluid, particle release and hydraulic gradient. There is an additional literature that focuses on the blocking effect of clay on inorganic substances and pollutants. However, research regarding how clay affects the downward intrusion of shallow saline groundwater and conventional ion blockage is still largely absent.

In this study, we analyzed the blocking effect of clay on Na^+^, Ca^2+^ and Mg^2+^ in groundwater using real-time monitoring of field water quality, adsorption isothermal experiments, factor analysis and correlation analysis. The results have shown that the formation of groundwater chemical components in inland plains is affected by cation exchange adsorption, acid-based evolution, mixing, carbonic acid evolution, concentration, dissolution/precipitation of calcite and gypsum, and leaching. The effects of precipitation and leaching on the quality of shallow saline water, however, are not obvious. It was also found that the optimal adsorption isotherms of clay for Na^+^, Ca^2+^ and Mg^2+^ in groundwater at different depths are in accordance with the Henry and Langmuir equations. The results show that clay has a blocking effect on Na^+^, which is consistent with the results of previous tests. However, the research results regarding the blocking effect of clay on Ca^2+^ and Mg^2+^ in groundwater are limited and make no comparison. Here we have found that the adsorption capacity of clay increases with an increase in the concentration of Na^+^, Ca^2+^ and Mg^2+^. We have also found that, as the concentration of ions increases, the adsorption capacity continues to increase, suggesting a rapid rate of change. The intensity of the blocking effect of clay on Na^+^ (Ca^2+^, Mg^2+^) varied according to depth and was controlled by the type and content of minerals, with clay having a strong capacity to retard Na^+^ at certain depths. We can conclude, in that case, that the blocking effect of clay influences the formation and zoning of water chemical components, further confirming that clay has a controlling effect on the downward movement of salt water. 

However, the formation of groundwater chemistry components is not only related to hydrogeochemistry, but also closely related to palaeogeographic climate, hydrodynamic conditions and stratum lithology. This study has only focused on the blocking effect that clay can have on Na^+^, Ca^2+^ and Mg^2+^ in groundwater. The controlling effect of clay on conventional anions and other factors affecting the formation of water chemistry still require further research.

## Figures and Tables

**Table 1:** Basic parameters for the hydrogeological drilling.

Hydrological Observation Borehole Number	WX01	WX02	WX03	WX04
Borehole depth/m	12.0	49.2	95.0	113.6
Filtration pipe depth/m	8.0–12.0	13.0–49.2	79.0–94.0	98.0–107.9
Lithology at the depth of the Filtration pipe	Silty soil, silty Clay clip silt	Silty clay, silty Soil, silty sand	Silty clay, Clay	Silt, fine sand, Medium sand

**Table 2:** Basic physical and chemical properties of the clay samples.

Clay Sampling Depth/m		6.0–6.2	48.0–48.2	51.0–51.2	71.0–71.2	84.0–84.2	97.0–97.2	102.0–102.2	112.0–112.2
Porosity Ratio		0.612	0.535	0.510	0.512	0.564	0.645	0.496	0.849
Mineral Amount/%	Quartz	55.0	34.3	-	36.2	47.4	52.6	21.1	37.7
	Calcite	0.0	12.6	-	16.7	8.3	1.1	54.9	25.7
	Feldspar	15.0	7.5	-	11.8	17	15.1	0.1	15.5
	Illite	8.0	35.8	-	31.2	24.4	29.5	14.5	13.5
	Montmorillonite	12.0	2.0	-	0.4	0.4	0.4	8.9	0.4
	Kaolinite	10.0	7.8	-	3.7	2.5	1.3	0.5	7.2

**Table 3:** Milliequivalent percentage statistics for the experimental water chemical composition (*n* = 392).

Test Composition	Cation Ratio/%				Anion Ratio/%		
	K^+^	Na^+^	Ca^2+^	Mg^2+^	Cl^−^	SO_4_^2−^	HCO_3_^−^
WX01	0.04	51.71	11.57	36.68	57.79	29.23	12.98
WX02	0.02	41.09	15.03	43.86	49.84	48.38	1.78
WX03	0.04	41.59	18.66	39.71	65.81	31.48	2.71
WX04	0.03	41.46	27.1	31.41	44.57	47.3	8.13

**Table 4:** Eigenvalues and cumulative contributions for the factor analysis.

Hydrological Observation Borehole	Main Ingredient	Initial Feature Value			Rotation Square and Load		
		Eigenvalues	Variance/%	Cumulative Variance/%	Eigenvalues	Variance/%	Cumulative Variance/%
WX01	1	5.775	64.164	64.164	5.775	64.164	64.164
	2	2.811	31.235	95.398	2.811	31.235	95.398
WX02	1	7.150	79.446	79.446	7.150	79.446	79.446
	2	1.525	16.941	96.388	1.525	16.941	96.388
WX03	1	3.145	34.943	34.943	3.145	34.943	34.943
	2	2.016	22.397	57.340	2.016	22.397	57.340
	3	1.211	13.456	70.797	1.211	13.456	70.797
WX04	1	4.918	54.647	54.647	4.918	54.647	54.647
	2	1.315	14.607	69.254	1.315	14.607	69.254
	3	1.092	12.138	81.393	1.092	12.138	81.393

**Table 5:** Rotational factor load matrix for the groundwater at different depths.

Water Chemistry Index	WX01		WX02		WX03			WX04		
	F1	F2	F1	F2	F1	F2	F3	F1	F2	F3
Na^+^	−0.135	0.973	0.984	0.174	0.908	−0.008	0.157	0.850	−0.121	−0.004
Ca^2+^	−0.992	0.109	0.967	−0.091	0.145	−0.504	0.620	0.701	0.425	−0.357
Mg^2+^	0.999	−0.017	0.971	0.174	−0.323	0.845	0.105	0.790	−0.172	0.456
HCO_3_^−^	−0.978	0.690	−0.780	0.563	0.524	0.308	0.295	0.671	−0.646	0.100
Cl^−^	0.965	0.257	0.974	0.190	0.242	−0.730	−0.317	−0.730	−0.091	0.235
SO_4_^2−^	0.863	0.469	0.946	0.273	0.654	0.295	0.410	0.901	0.121	−0.001
pH	−0.364	0.921	0.141	−0.988	0.689	0.462	−0.262	0.688	0.510	−0.237
EC	0.203	−0.825	−0.919	0.212	0.911	−0.250	−0.168	−0.052	0.610	0.765
Total Hardness	0.984	0.179	0.992	0.078	−0.361	−0.238	0.583	0.900	−0.134	0.224

Note: F1, F2, F3 represents main factors. EC represents conductivity.

**Table 6:** The adsorption isotherm model parameters of clay for Na^+^, Ca^2+^, Mg^2+^ in groundwater.

Clay Sampling Depth/m	Ion in Groundwater	Langmuir Equation Parameters			Henry Equation Parameters	
		*q_m_*/(mg·kg^−1^)	*K_L_*/(L·mg^−1^)	*R* ^2^	*K_d_*/(L·mg^−1^)	*R* ^2^
6	Na^+^	4.60	0.0011	0.9164	0.2912	0.6311
	Ca^2+^	1.87	0.0150	0.7438	0.1020	0.4001
	Mg^2+^	2.72	0.0269	0.0394	0.2396	0.3851
48	Na^+^	1009.09	0.0453	0.7150	0.8606	0.0783
	Ca^2+^	466.67	0.0036	0.9408	0.8339	0.8511
	Mg^2+^	600.00	0.0013	0.9853	0.8451	0.9637
51	Na^+^	22.37	0.0014	0.7528	1.0739	0.8907
	Ca^2+^	909.09	0.0006	0.7718	0.9191	0.7074
	Mg^2+^	833.33	0.0003	0.8889	0.8132	0.7468
71	Na^+^	1000.00	0.0004	0.4764	0.4855	0.8182
	Ca^2+^	15,873.02	0.0001	0.9229	0.6738	0.9463
	Mg^2+^	909.09	0.0005	0.9116	0.3701	0.7336
84	Na^+^	277.78	0.0023	0.2421	0.7254	0.1848
	Ca^2+^	1250.00	0.0002	0.9976	0.0262	0.8260
	Mg^2+^	1250.00	0.0003	0.5574	0.6570	0.9237
97	Na^+^	416.67	0.0006	0.9382	0.5368	0.9011
	Ca^2+^	222.22	0.0007	0.4554	0.1636	0.5880
	Mg^2+^	769.23	0.0008	0.5940	0.6876	0.8955
102	Na^+^	400.00	0.0019	0.8942	0.4518	0.7132
	Ca^2+^	625.00	0.0003	0.6343	0.2070	0.8226
	Mg^2+^	11,235.96	0.0001	0.9836	0.4606	0.9069
112	Na^+^	192.31	0.0012	0.6352	1.5095	0.9251
	Ca^2+^	5000.00	0.0001	0.9031	0.2111	0.5134
	Mg^2+^	3333.33	0.0001	0.9403	0.3774	0.8569

Note: *q_m_* is the maximum adsorption capacity of the adsorbent in the Langmuir adsorption equation (mg kg^−1^); *K_L_* is the adsorption equilibrium constant (L mg^−1^) of the adsorbent in the Langmuir adsorption equation; *R*^2^ is correlation coefficient.

**Table 7:** The retardation coefficient of clay for Na^+^, Ca^2+^ and Mg^2+^ in groundwater.

Sampling Depth/m	*R_d-Na_*	*R_d-Ca_*	*R_d-Mg_*	Sum of Retardation Coefficients
6	1.2474	1.1053	4.0255	6.3782
48	2.0168	1.9852	1.9852	5.9873
51	5.5907	3.3209	2.2281	11.1397
71	3.0675	3.869	2.8470	6.9365
84	3.4604	1.1012	3.5397	8.1013
97	2.8144	1.5529	3.3240	7.6913
102	2.9859	1.9099	3.0245	7.9203
112	4.8762	1.5420	1.9691	8.3873

Note: *R_d__-Na_* is the retardation coefficient of Na^+^. *R_d__-Ca_* is the retardation coefficient of Ca^2+^. *R_d__-Mg_* is the retardation coefficient of Mg^2+^.
